# A discontinuous Galerkin model for fluorescence loss in photobleaching of intracellular polyglutamine protein aggregates

**DOI:** 10.1186/s13628-018-0046-0

**Published:** 2018-11-29

**Authors:** Christian V. Hansen, Hans J. Schroll, Daniel Wüstner

**Affiliations:** 1 Department of Mathematics and Computer Science, University of Southern Denmark, Campusvej 55, Odense M, 5230 Denmark; 2 Department of Biochemistry and Molecular Biology, Campusvej 55, Odense M, 5230 Denmark

**Keywords:** Discontinuous Galerkin, FLIP, Protein aggregation, Rate coefficient, Multi-compartment, Computational method, Calibration

## Abstract

**Background:**

Intracellular phase separation and aggregation of proteins with extended poly-glutamine (polyQ) stretches are hallmarks of various age-associated neurodegenerative diseases. Progress in our understanding of such processes heavily relies on quantitative fluorescence imaging of suitably tagged proteins. Fluorescence loss in photobleaching (FLIP) is particularly well-suited to study the dynamics of protein aggregation in cellular models of Chorea Huntington and other polyQ diseases, as FLIP gives access to the full spatio-temporal profile of intensity changes in the cell geometry. In contrast to other methods, also dim aggregates become visible during time evolution of fluorescence loss in cellular compartments. However, methods for computational analysis of FLIP data are sparse, and transport models for estimation of transport and diffusion parameters from experimental FLIP sequences are missing.

**Results:**

In this paper, we present a computational method for analysis of FLIP imaging experiments of intracellular polyglutamine protein aggregates also called inclusion bodies (IBs). By this method, we can determine the diffusion constant and nuclear membrane transport coefficients of polyQ proteins as well as the exchange rates between aggregates and the cytoplasm. Our method is based on a reaction-diffusion multi-compartment model defined on a mesh obtained by segmentation of the cell images from the FLIP sequence. The discontinuous Galerkin (DG) method is used for numerical implementation of our model in FEniCS, which greatly reduces the computing time. The method is applied to representative experimental FLIP sequences, and consistent estimates of all transport parameters are obtained.

**Conclusions:**

By directly estimating the transport parameters from live-cell image sequences using our new computational FLIP approach surprisingly fast exchange dynamics of mutant Huntingtin between cytoplasm and dim IBs could be revealed. This is likely relevant also for other polyQ diseases. Thus, our method allows for quantifying protein dynamics at different stages of the protein aggregation process in cellular models of neurodegeneration.

**Electronic supplementary material:**

The online version of this article (10.1186/s13628-018-0046-0) contains supplementary material, which is available to authorized users.

## Background

Our understanding of protein transport and aggregation has been revolutionalized by the development of genetically encoded fluorescent protein tags combined with technical innovations in high-resolution live cell fluorescence imaging. In particular, various advanced imaging methods have been used to study aggregation and phase partitioning of proteins in the nucleus and cytosol. Such protein segregation and aggregation is a hallmark of various age-associated neurodegenerative diseases, such as Alzheimer’s disease, Chorea Huntington, Ataxia or Parkinson disease. In several inherited neurodegenerative diseases, like ataxia and Huntington disease, certain proteins bearing a CAG triplet expansion coding for an extended poly-glutamine (polyQ) stretch causes the affected proteins to show the tendency to self-associate and form small and large aggregates, the latter also called inclusion bodies (IBs).

Formation of IBs has been associated with disease progression, but it remains unclear, whether such large aggregates are cytoprotective or cytotoxic [1–3]. In Huntington disease, the polyQ protein is mutated huntingtin (mtHtt) containing more than 30 glutamine repeats typically, while in ataxia, one finds one out of various ataxin proteins mutated containing a polyQ stretch.

The aggregation process in Huntington disease and related polyQ diseases has been studied extensively.Typically, suitable model cells are transfected with fluorescent protein-tagged derivatives of the studied polyQ protein, and the aggregation process is studied by a variety of methods including photobleaching techniques like fluorescence recovery after photobleaching (FRAP) and fluorescence loss in photobleaching (FLIP) [4–7], number and brightness (N & B) analysis of intensity fluctuations [8], fluorescence complementation assays with split GFP [9], Förster resonance energy transfer (FRET) [4, 6, 10], fluorescence correlation spectroscopy [10], fluorescence lifetime microscopy [4, 11], fluorescence anisotropy imaging [12], stimulated emission depletion (STED) microscopy [13] or single molecule tracking (SMT) [13–15]. Using such techniques, different aspects of the aggregation process have been revealed. In particular, it has been suggested that diffusive oligomers and small fibrillary aggregates co-exist with IBs, which accumulate after some delay as clearly discernable micron-sized structures [8, 13, 16–18]. The oligomers or protein fibrils are sometimes difficult to detect, first due to their small size compared to IBs and second due to their low brightness which makes that they are often overshined by the much brighter IBs [8, 13, 15]. However, also the micron-sized IBs formed of green fluorescent protein–tagged mtHtt (GFP-mtHtt) come in strongly varying brightness levels and are eventually replaced by similarly sized but much more dynamic and eventually less bright intermediate structures in the aggregation process [13, 15]. Indeed, protein aggregates detected in cellular models of polyQ diseases are dynamic entities, often recruiting other proteins and thereby sequestering enzymes and signaling proteins which strongly affect the functionality of cells [5–7, 9]. In detailed FRAP and FLIP studies, both fast- and slow exchanging components have been described for ataxins and mtHtt with half-times for the exchange of tagged protein between cytoplasm and IBs in the range of less than 10-20 sec for various ataxins [19, 20] over 1-2 min for larger IBs of mtHtt6 [4, 20]. This strongly suggests that different populations of inclusions with different physico-chemical properties coexist in affected cells. Supporting that notion, both fibrillary and globular IBs have been detected upon expression of fluorescent protein–tagged mtHtt in the same cells, and this structural heterogeneity was reflected in differing exchange dynamics [4]. An additional level of complexity comes from the complex architecture of the cytoplasm, which generates sub-compartments of varying composition not only via membrane-bound organelles but also in the form of membrane-less liquid phases into which proteins can partition differently [21]. It has been suggested that such variety of physico-chemical phases in the cyto- and nucleoplasm can be a driving force for protein segregation, and in case of mutated polyQ proteins, trigger protein aggregation [22].

Aggregates of polyQ proteins can form in both, the cytoplasm and nucleus, and some polyQ proteins, such as mtHtt or ataxins have been shown to bear nuclear localization and export signals, suggesting active transport across the nuclear membrane [23–26]. On the other hand for mtHtt, a Ran-GTPase independent transport across the nuclear membrane has been described [27]. How the nucleo-cytoplasmic transport of polyQ proteins is kinetically coupled to their intracellular diffusion and binding to IBs is not known. FLIP is in principle an ideal method to answer this question, as fluorescence loss in different cellular areas can be quantified for repeated localized bleaching far from IBs. However, most studies applying FLIP in this context do not attempt to develop a physical model underlying the observed fluorescence loss kinetics [5, 6, 19]. In a previous study, we presented the first attempt at developing a quantitative FLIP model to estimate exchange rate constants for GFP-mtHtt from FLIP image sequences [7]. We tracked individual IBs and determined exchange rate constants relative to the overall fluorescence loss kinetics based on a multi-compartment model. However, this method lacked a proper description of intracellular diffusion and nucleo-cytoplasmic exchange of GFP-mtHtt not associated with the IBs [7]. In a separate study, we developed a reaction-diffusion model to quantify diffusion and nucleo-cytoplasmic exchange parameters for GFP as measured in FLIP experiments [28]. For that, we made use of a reaction-diffusion multi-compartment model implemented into FEniCS and solved that on a meshed surface geometry directly obtained from the cell images in the FLIP sequence. We used a discontinuous Galerkin (DG) model for improved boundary description and numerical integration of the underlying partial differential equation (PDE) system after transforming that into the weak form.

Here, we combine and extend both approaches and present what we believe is a new computational method to directly infer quantitative dynamic parameters for transport and aggregation of polyQ proteins in living cells. We suggest two modes of nucleo-cytoplasmic transport of GFP-mtHtt and determine diffusion constants and nuclear membrane coefficients as well as binding dynamics of GFP-mtHtt to IBs in concert with bleaching coefficients for the intended laser bleach in the FLIP experiment directly from experimental confocal FLIP images.

## Methods

### A reaction–diffusion model on real cell geometry.

In [28] we present a reaction–diffusion model with semipermeable nuclear membrane and hindrance for spatial heterogeneity. In this paper, the mathematical model is extended such that it can be applied to describe additionally protein aggregations from FLIP image sequences of living cells. Further, both the semipermeable model and also an active transport model for the nuclear membrane is presented. As described in [28] an appropriate FLIP model has to account for dynamic heterogeneity, local hindrance and molecular crowding in living cells, which are very conspicuous on the FLIP images. As in [28], it is assumed that the high-intensity areas are the areas in which we find that GFP-mtHtt is hindered in its motion. Therefore, our computational FLIP model allows for this by a space-dependent first order reaction given by:

1 $$ u \mathop{\rightleftharpoons}^{k_{\text{on}}}_{k_{\text{off}}} u_{b},  $$

where *u* and *u*_*b*_ are the intensities of the free and hindered molecules, respectively.

The observed fluorescence intensity from the FLIP images is described by:

2 $$  c = u + u_{b}.  $$

For areas with high intensity we would find a higher population of the hindered *u*_*b*_ proteins. Then given the first order reaction kinetic (1), the space dependent reaction rate *k*_on_ will be high in high-intensity areas and zero in the areas with lowest intensities. First assume that the first FLIP image is in equilibrium and the free molecules are uniformly distributed, next let *c*^0^ be the observed intensity from the first FLIP image, *u*^0^ be the intensity of the free molecules and $u_{b}^{0}$ be the intensity of the hindered molecules such that (2) is fulfilled. Letting *γ* be the proportionality constant then by [28] the reaction rates are set to:

3 $$  k_{\text{on}}(\mathbf{x}) = \gamma u_{b}^{0}(\mathbf{x}) = \gamma \left(c^{0}(\mathbf{x}) - u^{0} \right) \enspace,  $$

where *γ* is a proportionality constant. Consequently, *k*_off_ is constant

4 $$  k_{\text{off}} = \frac{k_{\text{on}}(\mathbf{x})}{u_{b}^{0}(\mathbf{x})} u^{0} = \gamma u^{0} \enspace.  $$

Letting diffusion be expressed in the terms of Fick’s law and *α* being the diffusion constant for the free molecules, our time-dependent PDE model reads:

5 $$\begin{array}{*{20}l}   u_{t} &=\left. \nabla \cdot (\alpha \nabla u) + k_{\text{off}}u_{b} - k_{\text{on}}u- \theta b \frac{q}{1+q}u \right\vert_{\Omega_{B}} \enspace,\\ (u_{b})_{t} &= \left. k_{\text{on}}u - k_{\text{off}}u_{b} - \theta b \frac{q}{1+q}u_{b} \right\vert_{\Omega_{B}} \enspace,\\{} & \quad \mathbf{x} \in \Omega \enspace, \quad t > 0 \enspace, \end{array} $$

where *θ* is the time dependent indicator function simulating the high intensity laser bleaches, *b* is the intrinsic bleaching rate constant, *q* is the equilibrium constant for the reaction between the ground and excited state for a fluorophore [29] and *u*_*t*_ is the time derivative of *u*. For mass conservation the Neumann boundary condition along *∂Ω* is used,

6 $$  \mathbf{n}\cdot \nabla u = \mathbf{n}\cdot \nabla u_{b} = 0 \enspace, \quad \mathbf{x} \in \partial \Omega \enspace,  $$

where **n** is the outward unit normal. With initial conditions:

7 $$  u(0,\mathbf{x}) = u_{0}(\mathbf{x}) \enspace, \quad u_{b}(0,\mathbf{x}) = (u_{b})_{0}(\mathbf{x}) \enspace,\quad \mathbf{x} \in \Omega \enspace.  $$

Next, two different membrane models are suggested.

### Permeable membrane model

For the semipermeable membrane model the cytoplasm and nucleus are separated by the nuclear membrane *Γ*_*M*_ with diffusive transport for GFP-mtHtt through the nuclear pore complex leading to the method presented in [28], where the diffusive flux is expressed as interface condition

8 $$  \mathbf{J} \cdot \mathbf{n}^{-} = -\alpha\frac {\partial u^{-}}{\partial \mathbf{n}^{-}} = p \llbracket u \rrbracket \mathbf{n}^{-} \quad \mathbf{x} \in \Gamma_{M} \enspace.  $$

Here, *p* is the permeability of the membrane measured in *μm*/*s*. The ± superscripts indicate that the parameter is measured in two adjacent triangles, and thus **n**^−^ is the outward normal for the triangle marked with the minus sign. As the outward normals along the common interface are opposite, consequently the flux in (8) is written as a jump bracket *⟦u⟧*=*u*^+^**n**^+^+*u*^−^**n**^−^. In this special case, the two adjacent triangles are placed with the nuclear membrane as their common edge. Consequently, one is located in the cytoplasm and one in the nucleus.

### Active transport - membrane model

**Fig. 1:**
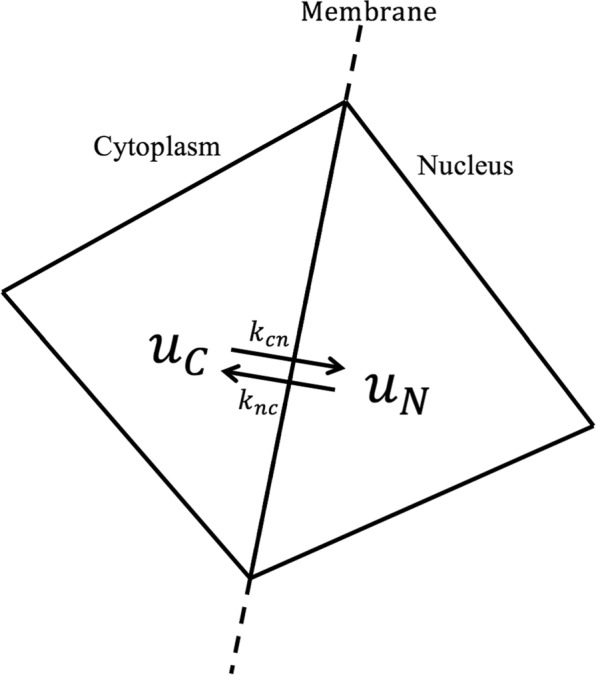
Transport kinetics for the active transport model across the nuclear membrane

Alternatively, we describe the nucleo-cytoplasmic transport of GFP-mtHtt as an active process. For that, we extend our previous model and include a reaction term across the nuclear membrane as shown in Fig. 1 and given in (10), below. This reaction term with different rate constants in both directions simplifies the known importin/exportin-mediated nuclear transport. It is known that differing concentration of the GDP- and GTP-bound form of the RanGTPase in the nucleus and cytoplasm control net accumulation of protein cargo in either compartment [30]. Thus, protein cargo is assumed to shuttle rapidly back and forth, but net accumulation is a consequence of the differing abundance of certain binding partners in both compartments [30–33]. Our model, thus, only accounts for the net kinetic effect of the transport machinery in the form of differing overall import and export rate constants for GFP-mtHtt. As illustrated in Fig. 1 which is a closeup view of our new membrane transport model, *u*_*C*_ and *u*_*N*_ are the intensities in each of the illustrated neighboring triangles, which are located at the cytoplasmic and nuclear side of the membrane, respectively. Thus, the first order reaction equation between the two triangles can be written as:

9 $$ u_{C} \mathop{\rightleftharpoons}^{k_{cn}}_{k_{nc}} u_{N}.  $$

Thus the PDE reads:

10 $$\begin{array}{*{20}l}  (u_{C})_{t} &= k_{nc} u_{N} - k_{cn} u_{C} \enspace,\\ (u_{N})_{t} &= k_{cn} u_{C} - k_{nc} u_{N} \quad \mathbf{x} \in \Gamma_{M} \ .  \end{array} $$

This reaction only happens between two adjacent triangles where their common edge is a part of the membrane line. An important property is that summing the two equations from (10) shows mass conservation.

### Multi-compartment modeling of GFP-mtHtt exchange

In [7] a simple multi-compartment model was developed to describe exchange of GFP-mtHtt between cytoplasm and aggregates. The multi-compartment approach is here implemented into the reaction-diffusion FLIP model as an internal interface conditions, with the first order transport kinetics described as:

11 $$ u_{C} \mathop{\rightleftharpoons}^{k_{1}}_{k_{2}} u_{A},  $$

where *u*_*C*_ is the intensity in the cytoplasm and *u*_*A*_ is the intensity in the respective aggregate. Contrary to (1), which by hindrance organize spatial heterogeneity in the full cell, (11) is used to describe the exchange between cytoplasm and aggregates and thereby form multiple compartments. Expressed as a differential equation the mass preserving transport process becomes:

12 $$\begin{array}{*{20}l}  (u_{C})_{t} &= k_{2} u_{A} - k_{1} u_{C} \enspace,\\ (u_{A})_{t} &= k_{1} u_{C} - k_{2} u_{A} \ .  \end{array} $$

**Fig. 2:**
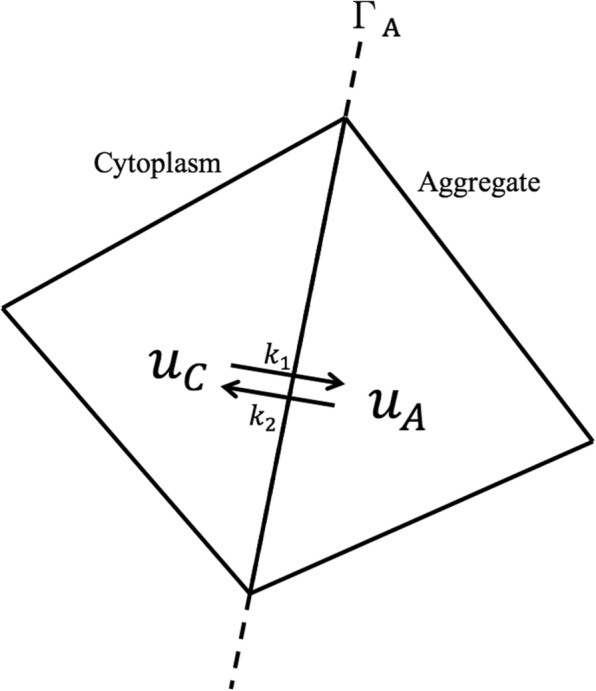
Transport kinetics between the aggregates and cytoplasm

As for the active membrane model presented above, these equations are now applied as an interface condition, *u*_*C*_ and *u*_*A*_ becomes the intensities in each of the illustrated neighboring triangles in Fig. 2, which are located at the cytoplasmic and aggregate side of the aggregates boundary *Γ*_*A*_, respectively.

Therefore this reaction only happens between two adjacent triangles where their common edge is a part of the line that separates the cytoplasm and aggregates.

### Cell geometry

**Fig. 3:**
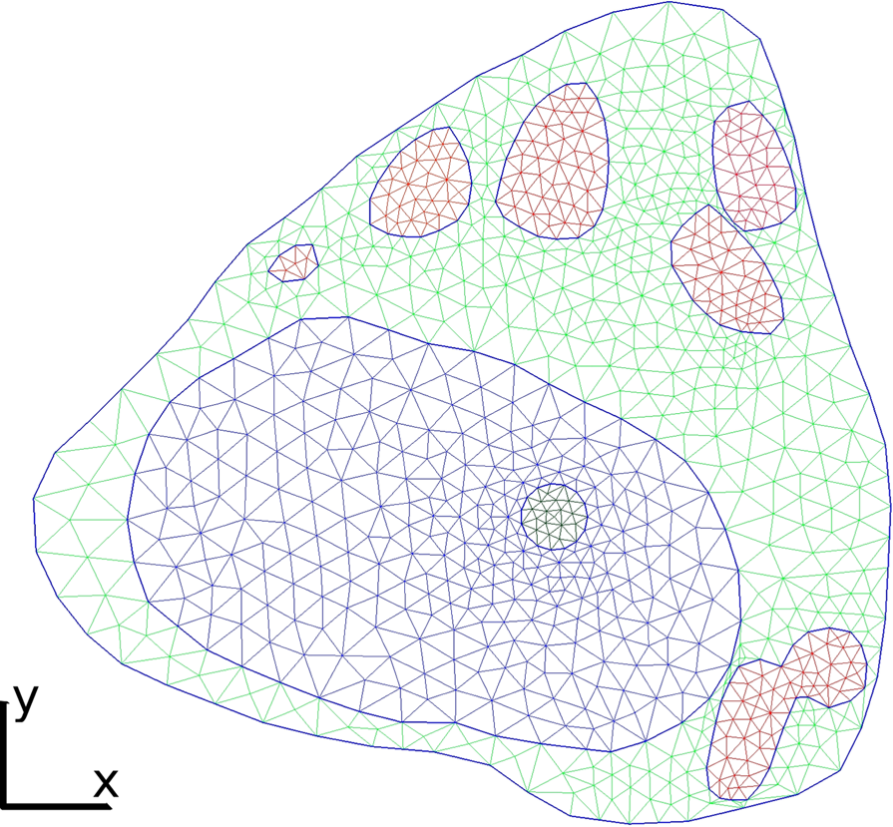
Mesh with 1825 triangles on the real cell geometry. The green triangles constitute the cytoplasm, in red is the aggregates, the dark blue triangles form the nucleus and inside nucleus the round bleaching area with a diameter of 25 pixel can be found

The cell geometry (see Fig. 3) is like in [28, 34] conveyed from the FLIP images by use of an extended implementation of [35] which uses the "Active Contours Without Edges" method by Chan and Vese [36]. The Chan-Vese model does not depend on the image gradients and is, therefore, able to accomplish a segmentation on more blurred images. The cell geometry is segmented from the first image whereas the aggregates are all segmented from the last FLIP image. As bleaching of the FLIP images occurs in the nucleus, it is hard to segment the nucleus automatically from the FLIP sequence. Thus the geometry of the nucleus is here set by hand. The mesh is generated on the geometry in Fig. 3 with Gmsh and then converted to XML-file.

### A discontinuous Galerkin method with internal interface condition

In [28], the interface condition along the nuclear membrane (8) was implemented into the IPDG method based on [37, 38]. Additionally, in this paper, the internal interface condition along the aggregate boundaries are implemented. To derive the weak formulation, we first consider the aggregate interface conditions.

Let the discretization of *Ω* be denoted by $\mathcal {T}_{h}$ consisting of disjoint open elements $\mathcal {K} \in \mathcal {T}_{h}$. While integrating along *Γ*_*A*_, *u*^−^ and *u*^+^ are considered as the values of two different but adjacent elements $\mathcal {K}^{+}$ and $\mathcal {K}^{-}$ with a common edge on *Γ*_*A*_. To rewrite (12) into integral form with the *u*^−^ and *u*^+^ notation, (12) is split up in two cases, one if *u*^−^ is in the cytoplasm and one if *u*^−^ is in the aggregate. An indicator function *I*_*C*_ is therefore introduced as:

13 $$ I_{C}(u) = \left\{ \begin{array}{ll} 1 & \text{if}\ \mathcal{K}_{u} \in \Omega_{C}\\ 0 & \text{else.} \end{array}\right.   $$

Thus the weak form reads:

14 $$ \int_{\Omega} u_{t}\ \mathrm{d}x = A(u,v) \,  $$

where

15 $$\begin{array}{*{20}l} {} A\!(u,\!v)\!\! &:=\!\!\! \int_{\Gamma_{A}} \!I_{C}(u^{+})\!\! \left(\left(k_{2}u^{-}\,-\,k_{1}u^{+}\right)\!v^{+} \,+\, \left(k_{1}u^{+}\!\,-\,k_{2}u^{-}\right)\!v^{-} \!\right)\ \!\!\mathrm{d}S \\ &\;\;+\!\! \int_{\Gamma_{A}} \!\!I_{C}(u^{-})\!\! \left(\left(k_{1}u^{-}\!\,-\,k_{2}u^{+}\right)\!v^{+} \,+\, \left(k_{2}u^{+}\!\,-\,k_{1}u^{-}\right)\!v^{-} \!\right) \ \!\!\mathrm{d}S  \end{array} $$

and *v* as the usual test function.

For notation, now let *Γ* denote the union of the boundaries of all the disjoint open elements $\mathcal {K}$. Furthermore, let *Γ* consist of four disjoint subsets, such that *Γ*=*∂Ω*∪*Γ*_int_∪*Γ*_*M*_∪*Γ*_*A*_. Thus *Γ*_int_ holds all internal edges. Then usual average and jump term for DG-methods are defined as {*u*}=(*u*^+^+*u*^−^)/2, *⟦u⟧*=*u*^+^**n**^+^+*u*^−^**n**^−^. For vector valued functions **q** the average and jump term are defined as: {**q**}=(**q**^+^+**q**^−^)/2, *⟦***q***⟧*=**q**^+^·**n**^+^+**q**^−^·**n**^−^. where **n**^±^ is the outward unit vectors on $\partial \mathcal {K}^{\pm }$.

Reusing the notation from [28] we let

16 $$\begin{array}{*{20}l} D(u,v,\alpha) :=& \int_{\Omega} \alpha \nabla u \cdot \nabla v\ \mathrm{d}x - \int_{\Gamma_{\text{int}}} \{ \alpha \nabla v \} \cdot \llbracket u \rrbracket\ \mathrm{d}s  \\ & -\!\! \int_{\Gamma_{\text{int}}} \{\alpha \nabla u \} \!\cdot\! \llbracket v \rrbracket\ \mathrm{d}s +\!\! \int_{\Gamma_{\text{int}}} \frac{\sigma}{h} \llbracket u \rrbracket \cdot \llbracket v \rrbracket\ \mathrm{d}s \enspace \!\!, \end{array} $$

17 $$\begin{array}{*{20}l} R(u,u_{b},v) :=& \int_{\Omega} (k_{\text{off}}u_{b} - k_{\text{on}}u)v\ \mathrm{d}x \, \end{array} $$

18 $$\begin{array}{*{20}l} B(u,v) :=& \int_{\Omega_{B}} \theta b \frac{q}{1+q}u v\ \mathrm{d}x \ .  \end{array} $$

Thus our weak formulation reads:

19 $$ \begin{aligned} &\int_{\Omega} u_{t} v \ \mathrm{d}x + D(u,v, \alpha) = \ R(u,u_{b},v) \\&\qquad\qquad\quad- B(u,v) + A(u,v) + M(u,v) \, \\  &\int_{\Omega} (u_{b})_{t} w \ \mathrm{d} x = -R(u,u_{b},w) - B(u_{b},w) \enspace, \end{aligned}  $$

where *v* and *w* are the usual test functions. *M*(*u*,*v*) represent the transport mechanism for the chosen membrane model.

For the semipermeable membrane model let:

20 $$ M(u,v) := - p \int_{\Gamma_{M}} \llbracket u \rrbracket \cdot \llbracket v \rrbracket \ \mathrm{d}s \ .  $$

The weak form for the active membrane model reads

21 $$ \begin{aligned} M(u,v) &:= \int_{\Gamma_{M}} I_{C}\left(u^{+}\right) \left(\left(k_{nc}u^{-}-k_{cn}u^{+}\right)v^{+}\right.\\ &\quad\left. + \left(k_{cn}u^{+}-k_{nc}u^{-}\right)v^{-} \right) \ \mathrm{d}S \\ &\quad+\int_{\Gamma_{M}} I_{C}(u^{-})\left(\left(k_{cn}u^{-}-k_{nc}u^{+}\right)v^{+} \right.\\ &\quad\left. +\left(k_{nc}u^{+}-k_{cn}u^{-}\right)v^{-} \right) \ \mathrm{d}S. \end{aligned}   $$

Any L-stable method can be used for discretizing the time derivative. Here the backward Euler is used for the implementation using the automated Finite Element package FEniCS [39]. Pre–assemble the system matrix will improve the computational time in FEniCS. However, as the bleaching term is time dependent the system is here pre–assembled into two system matrices. One with and one without the bleaching term. Inside the python script, the weak formulation is therefore expressed twice in the UFL form language, however, in the short python script presented here, only the weak formulation from (19) which includes the bleaching term can be found.

For simplicity the bleaching term $b\frac {q}{1+q}$ from (18) is replaced by *β* in the implementation and calibration.


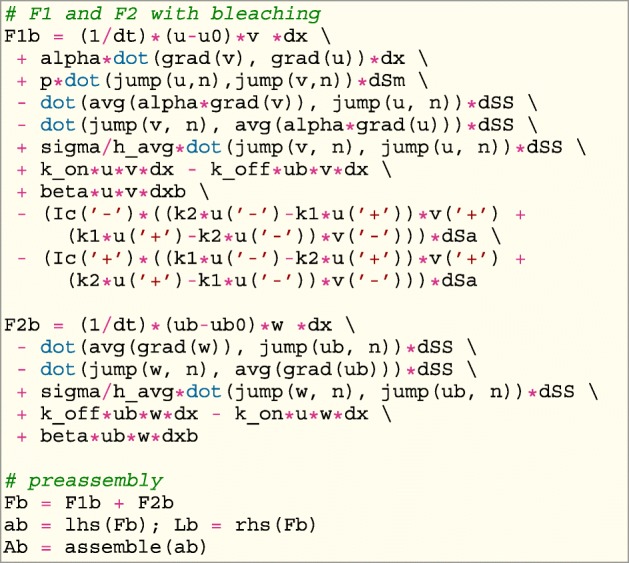


Where dSm represent the integral along the membrane, dSa is the integral along the aggregates boundaries, dSS is the integral on the remaining edges with smooth solutions and dxb represents the bleaching area. A similar system matrix is implemented without the bleaching term and the left-hand side is pre–assembled as the matrix A with the right-hand side L. The time dependent system is solved in FEniCS by:


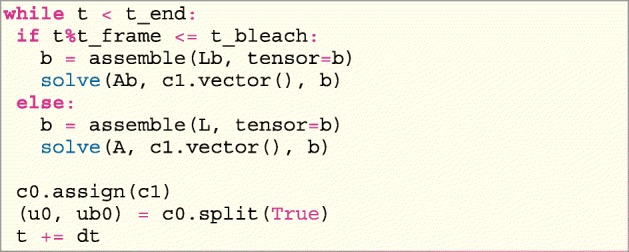


## Results

### Calibration and simulation of intracellular transport with the permeable membrane model

**Fig. 4:**
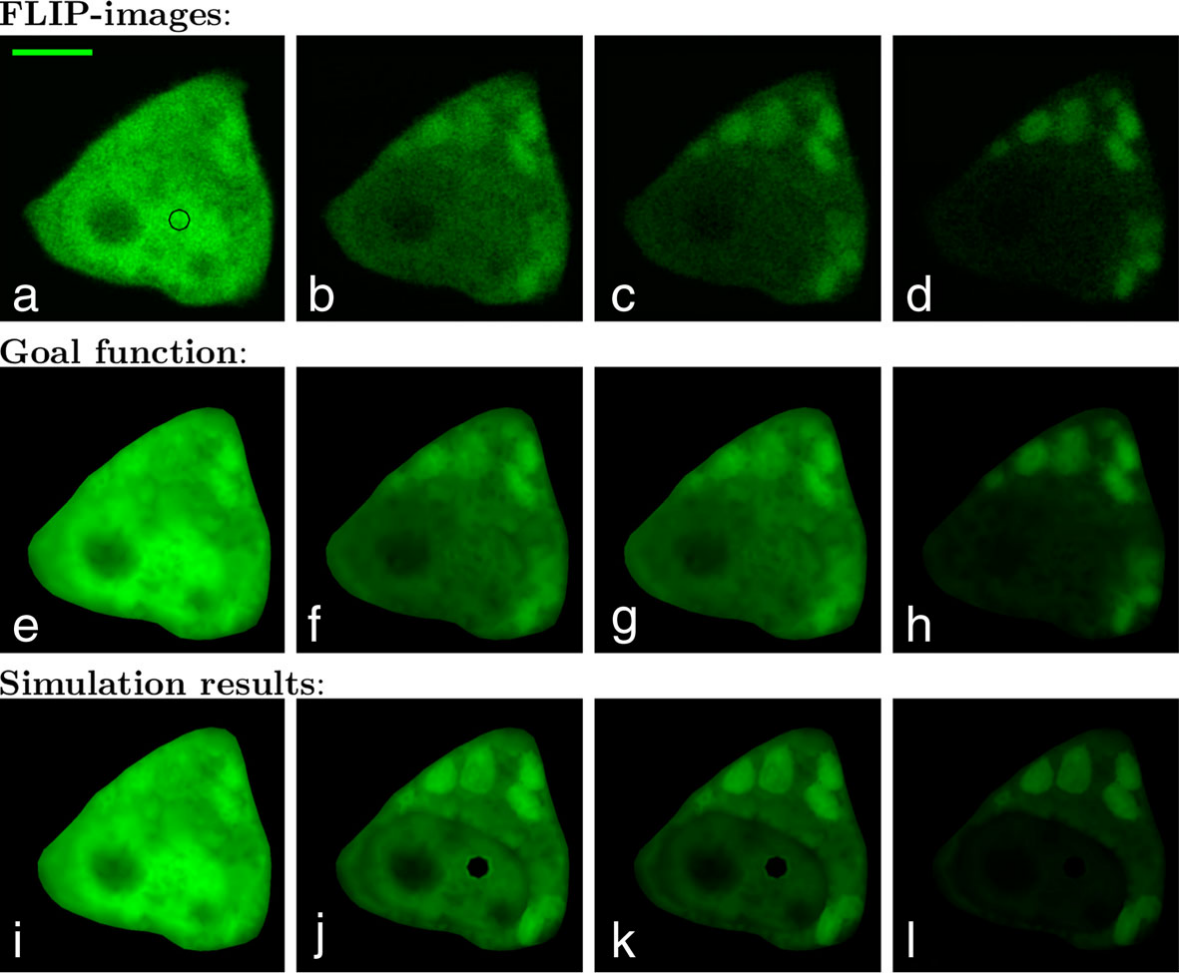
The first four images **a**-**d** are the original FLIP images of the CHO cells expressing GFP-Q73 in the cytoplasm and nucleus. It is produced in a temperature controlled (35±1°*C*) environment on a Zeiss LSM 510 confocal microscope using the 488nm line of an Argon laser. The black circle on the image **a** shows the 25-pixel wide bleaching area and a scalebar which is 5 *μ*m. The pixel size is here 0.0461847 *μ*m given a width of the bleach spot of ca. 1.15 *μ*m. The leftmost FLIP image **a** is taken before bleaching, the next image **b** is taken after it has been bleached 10 times, i.e., time *t*=28 s. The third FLIP image **c** is the 20’th FLIP image in the sequence (time *t*=56 s) and the last **d** is at time *t*=109.2 s which correspond to FLIP fame 39. The second row **e**-**h** shows the corresponding goal function. The third row **i**-**l** shows the simulation results, all at times corresponding to the displayed FLIP images

To calibrate the unknown parameters *α*,*β*,*γ*,*p*,*k*_1_,*k*_2_ we make a comparison between the simulation result and the FLIP images. The frame time for the FLIP experiment in Fig. 4a-d where *Δt*_*frame*_=2.8s, within that time the bleaching area with a diameter of 25*μ*m was bleached with 100% laser intensity for 2s. Thus the imaging process with a laser power of 0.5*%* took 0.8s.

To easily compare the simulation results and the FLIP sequence, the goal function seen in Fig. 4e-h is created. The goal function is a piecewise linear discontinuous Galerkin function defined on the mesh, which represents the values from the denoised FLIP images. To denoise the FLIP sequence, Gaussian blur with a radius of 1 pixel is used. At the discrete times *t*_*i*_=*Δt*_*frame*_(*i*−1)+*t*_*compare*_ seconds *i*=1,2,3,…,*n* the L_2_ norm of the difference between the goal function and the simulation is calculated to represent the misfit functional as:

22 $$  E = \frac{1}{n} \sum\limits_{i=1}^{n} \int_{\Omega} |u(t_{i},\mathbf{x})+u_{b}(t_{i},\mathbf{x}) - c_{g}(t_{i},\mathbf{x})|^{2}\ \mathrm{d}x \enspace,  $$

where *c*_*g*_ is the goal function. For the sequence in Fig. 4 the number of FLIP images is *n*=40 and the time where the simulation and FLIP data are compared is *t*_*compare*_=2.6s. To calibrate the unknown parameters, the Nelder–Mead downhill simplex algorithm [40] from the SciPy library [41] is used. The stop criterium is set such that either the difference in the parameter or the difference in the misfit functional between each iteration should be lower than 10^−4^. Looking at the reactions rates *k*_1_ and *k*_2_ it is known from (12) that in equilibrium the equilibrium constant can be described as:

23 $$ K = \frac{k_{1}}{k_{2}} = \frac{u_{A}}{u_{C}} \ .  $$

Assuming that the first FLIP image before bleaching (see Fig. 4a) is in equilibrium, *K* can be determined by the use of the average intensities from inside the aggregates and cytoplasm, respectively. From the FLIP image in Fig. 4a the equilibrium constant turns out to be *K*=1.16. Thus by expressing *k*_2_ in terms of *k*_1_, the parameters that need to be calibrated are reduced to *α*,*β*,*γ*,*p*,*k*_1_. The initial guesses for the calibration are set to *α*_0_=25, *β*_0_=20, *γ*_0_=0.5, *p*_0_=0.05 and (*k*_1_)_0_=0.001. After 405 iterations and 679 evaluations, the resulting calibrated parameters are

24 $$\begin{array}{*{20}l}  \widetilde{\alpha} &= 17.6 \ \mu\mathrm{m}^{2}/\mathrm{s} \enspace, \quad \widetilde{\beta} = 36.0 \text{ s}^{-1} \enspace,\\{} \quad \widetilde{\gamma} &= 0.198 \text{ s}^{-1} \enspace,\quad \widetilde{p} = 0.318 \ \mu\mathrm{m}/\mathrm{s} \, \\ \widetilde{k_{1}} &= 0.0718 \text{ s}^{-1} \, \quad \text{ and} \quad \widetilde{k_{2}} = \frac{\widetilde{k_{1}}}{1.16} = 0.0619 \text{ s}^{-1} \ .  \end{array} $$

The misfit functional with the initial parameters *E*_0_=7,141 was lowered to *E*=2,807 for the calibrated parameters in (24). The calibration process took around 9 h on an Intel Core i5 processor at 3.2 GHz with 8 GB memory running Ubuntu 16.04 LTS. The mesh used is presented in Fig. 3 and consists of 1825 triangles. The results of the calibration process are presented in Fig. 4i-l.

**Fig. 5:**
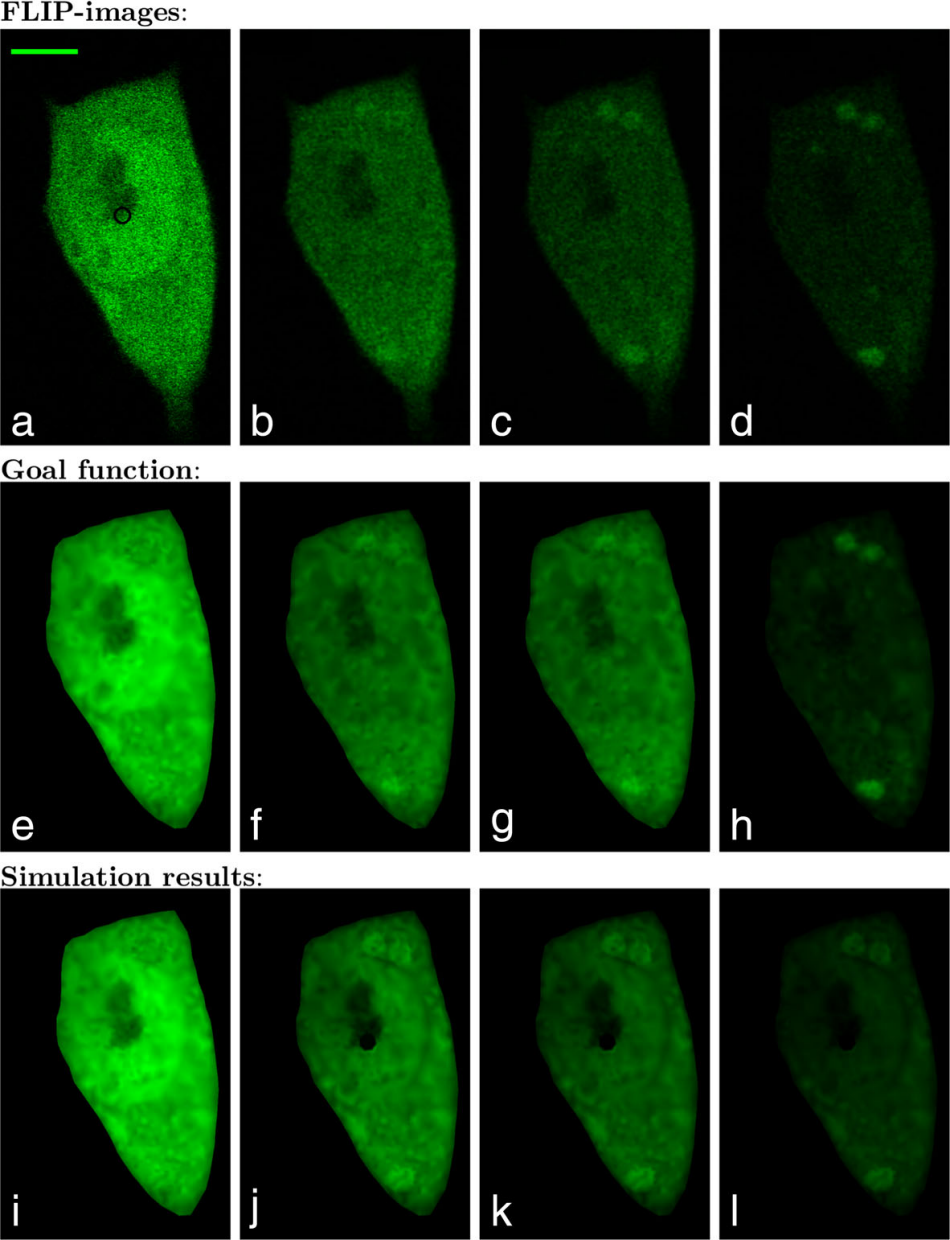
(**a**-**d**) are the original FLIP images of the CHO cells expressing GFP-Q73 in the cytoplasm and nucleus. The black circle on the image **a** shows the 18-pixel wide bleaching area in the nucleus with a pixel size of 0.0624404 *μ*m and a scalebar which is 5 *μ*m. **a** is taken before bleaching, **b** is after 10 time bleaches, i.e., time *t*=26 s. **c** is the 20’th FLIP image in the sequence (time *t*=52 s) and **d** is produced at time *t*=104 s which correspond to FLIP fame 40. The second row **e**-**h** shows the corresponding goal function. The third row **i**-**l** shows the simulation results, all at times corresponding to the displayed FLIP images

In Fig. 5a-d a similar FLIP sequence with *Δt*_*frame*_=2.6 s, *t*_*compare*_=2.4 s and *n*=55 can be seen. The simulations have been made on a mesh consistent of 1998 triangles, and the initial guesses for the calibration are set to *α*_0_=15, *β*_0_=10, *γ*_0_=0.05, *p*_0_=0.5 and (*k*_1_)_0_=0.01. After 353 iterations and 594 evaluations within 13 h the resulting calibrated parameters are

25 $$\begin{array}{*{20}l}  \widetilde{\alpha} &= 15.9 \ \mu\mathrm{m}^{2}/\mathrm{s} \enspace, \; \widetilde{\beta} = 34.6 \text{ s}^{-1} \enspace, \; \widetilde{\gamma} = 0.0614 \text{ s}^{-1} \enspace,\\\quad \widetilde{p} &= 0.447 \ \mu\mathrm{m}/\mathrm{s} \, \\ \widetilde{k_{1}} &= 0.0111 \text{ s}^{-1} \, \quad \text{ and} \quad \widetilde{k_{2}} = \frac{\widetilde{k_{1}} }{1.02}= 0.0109 \text{ s}^{-1} \ .  \end{array} $$

The misfit functional was lowered from *E*_0_=5,591 to *E*=2,531 during the calibration. The simulation result with the calibrated parameters can be seen in Fig. 5i-l.

### Active transport of GFP-mtHtt across the nuclear membrane

Inspired by our previous work on modeling FLIP data of GFP, we have used a semi-permeable model for nucleo-cytoplasmic transport of GFP-mtHtt so far. However, it turns out that the determined membrane permeability, *p*, of around 0.5 (see (24) and (25)), is very high for a protein, the size of ca. 2 GFP molecules, from which we determined previously a reasonable value of *p*=0.111 [28]. Thus, the relatively high permeabilities of the GFP-mtHtt protein may indicate that the traffic across the nuclear membrane could be caused by enzyme-catalyzed active transport [42]. Two to three days after transient transfection, we often observed slowed nuclear-cytoplasmic exchange of GFP–mtHtt compared to GFP, likely due to the pronounced formation of sub-resolution aggregates which interfere with normal nucleo–cytoplasmic transport (not shown but see Fig. 6 in [7]). Such varying results have been reported previously [27, 43–46] and they could be well attributed to the eventual occurrence of soluble oligomers, whose transport across the nuclear membrane is delayed, while transport of monomeric mtHtt profits from interaction with FG-rich repeats in the nuclear pore, which can accelerate transport compared to passive cargo [47].

**Fig. 6:**
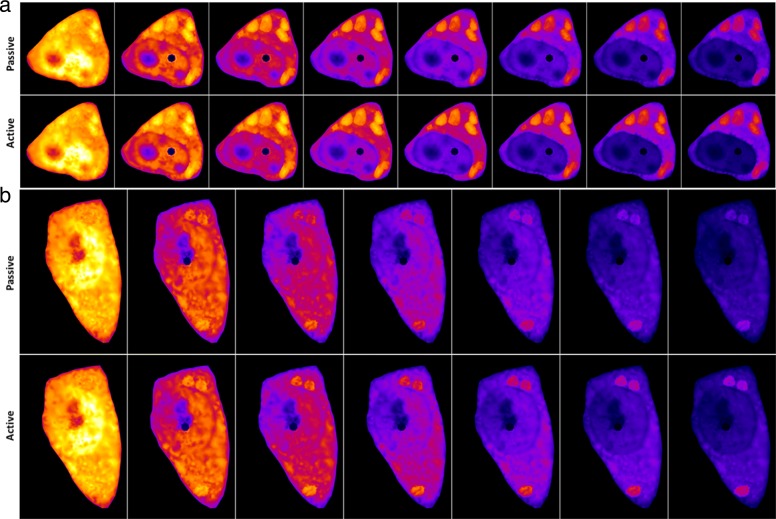
Simulation results for both type of models. **a** and **b** shows two different cells, the top row in both **a** and **b** shows the simulation results for the semi-permeable, whereas the second row displays the simulation results for the active membrane model. All are simulation results with the respectively calibrated parameters. The yellow areas are high-intensity areas, whereas the blue areas are low-intensity areas

To account for the possibility of active transport of GFP-mtHtt across the nuclear membrane, we have developed an alternative description of this transport step. The complex nuclear transport machinery was simplified by including unidirectional rate constants across the nuclear membrane (from the nucleus to the cytoplasm, *k*_*nc*_, and from cytoplasm to nucleus, *k*_*cn*_). These rate constants were determined directly from the FLIP data.

By the assumption that the first FLIP image is in equilibrium, it is possible to find the equilibrium constant *K*_*M*_ for *k*_*nc*_ and *k*_*cn*_, by measuring the average intensities inside the nucleus and the cytoplasm from the first FLIP image, respectively. Thus *k*_*nc*_ can be expressed as $k_{nc} = \frac {k_{cn}}{K_{M}}$, consequently only *k*_*cn*_ have to be calibrated. Thus for the active model, the parameters which needs to be calibrated are *α*,*β*,*γ*,*k*_1_,*k*_*cn*_. The two cells presented in the previous section are again used for calibration with the same boundary conditions and initial values, except for *p*_0_ which is replaced by (*k*_*cn*_)_0_=0.05, in both calibrations. Both calibrations are run on the same two meshes used for the permeable model. After 493 iterations, 828 function evaluations and 21 h of calibration the parameters for the first cell were found to be:

26 $$ \begin{aligned} \widetilde{\alpha} &= 19.4 \ \mu\mathrm{m}^{2}/\mathrm{s} \enspace, \quad \widetilde{\beta} = 52.7 \text{ s}^{-1} \enspace,\\ \quad \widetilde{\gamma} &= 0.156 \text{ s}^{-1} \enspace, \quad \widetilde{k_{1}} = 0.0556 \text{ s}^{-1} \, \\ \widetilde{k_{2}} &= \frac{\widetilde{k_{1}}}{1.16} = 0.0480 \text{ s}^{-1}, \quad \widetilde{k_{cn}} = 0.252 \text{ s}^{-1} \quad \text{ and} \quad \\ \widetilde{k_{nc}} &= \frac{\widetilde{k_{cn}}}{1.26} = 0.200 \text{ s}^{-1} \ .  \end{aligned}  $$

The the misfit functional for the initial values *E*_0_=6,277 was lowered to *E*=2,755 during the calibration. For the second cell the calibration took approximately 14 h to do 250 iterations and 434 function evaluations, resulting in the following parameter estimates:

27 $$\begin{array}{*{20}l} \widetilde{\alpha} &= 18.2 \mu\mathrm{m}^{2}/\mathrm{s} \enspace, \; \widetilde{\beta} \,=\, 32.0 \text{ s}^{-1} \enspace, \; \widetilde{\gamma} \,=\, 0.0617 \text{ s}^{-1} \enspace \!\!,\\\quad \widetilde{k_{1}} &= 0.0111 \text{ s}^{-1}, \\ \widetilde{k_{2}} &= \frac{\widetilde{k_{1}}}{1.02} = 0.0108 \text{ s}^{-1}, \quad \widetilde{k_{cn}} = 0.377 \text{ s}^{-1} \quad \text{ and} \\\quad \widetilde{k_{nc}} &= \frac{\widetilde{k_{cn}}}{1.10} = 0.342 \text{ s}^{-1} \ .  \end{array} $$

With a reduction in the misfit functional from *E*_0_=8,925 to *E*=2,527.

We directly compared the calibration results for both models in Fig. 6. Overall, the difference is minor, meaning that both models, i.e., with a passive exchange or active transport of GFP-mtHtt across the nuclear membrane can describe the experimental FLIP data equally well. In Fig. 6b there is a slightly more pronounced intensity in the IB’s for the active compared to the passive transport model. Since the exchange rate constants *k*_1_ and *k*_2_ are comparable, this could be a consequence of the slightly faster diffusion of GFP-mtHtt in the active transport model, making that the cytoplasmic signal surrounding the IBs decays faster compared to what is found in the passive model.

**Fig. 7:**
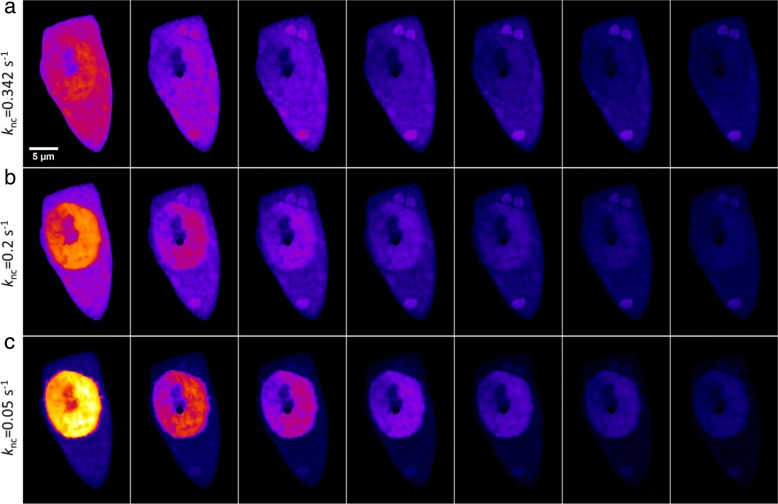
Simulation results illustrating an inhibition of nuclear export by lowered rate constant *k*_*nc*_. The three different simulations **a**, **b** and **c**, are all results of simulations of the model with the active membrane term. Common for them is that the parameters *α*,*β*,*γ*,*k*_1_,*k*_2_,*k*_*cn*_ are given from the calibration results in (27), wheres as *k*_*nc*_ is varying. First simulation **a** shows the resulting simulation from the calibration, i.e. *k*_*nc*_=0.342. In **b** nuclear export rate *k*_*nc*_ is lowered to 0.2 s^−1^. In **c** it is further lowered to *k*_*nc*_=0.05 s^−1^

The nuclear import and export rate constants we determined for GFP-mtHtt in both cells are in the same range with a slightly higher import than export rate constant (compare (26) and (27)). This reflects the experimental observation, that GFP-mtHtt does not become enriched in the nucleus compared to the cytoplasm. Huntingtin contains a conserved nuclear export signal (NES), and its export from the nucleus can be inhibited by mutations in the this NES or by using the inhibitor leptomycin B [26]. We simulate the effect of such an inhibition of nuclear export on FLIP image data of GFP-mtHtt by systematically lowering the rate constant *k*_*nc*_ in the active transport model Fig. 7. Our simulations predict that the lower *k*_*nc*_ is, the faster decays fluorescence of GFP-mtHtt in the nucleus when the bleach spot is located in this compartment. When *k*_*nc*_ is lowered more than 6 fold compared to control conditions (i.e. *k*_*nc*_=0.05 s^−1^ instead of *k*_*nc*_=0.342 s^−1^), we observe a strong accumulation of GFP-mtHtt in the nucleus and only very little enrichment in the cytoplasmic aggregates, see Fig. 7. This prediction could be directly tested in future experiments.

### Calibration test

To validate the calibration approach, a ground-truth in the form of a forward simulation of the semi-permeable membrane model with known parameters is made to represent and replace the FLIP images, which we calibrated against. The forward simulation is made with the same initial and boundary conditions as used in Fig. 4, on the mesh from Fig. 3. The chosen parameters are:

28 $$\begin{array}{*{20}l}  \alpha &= 17 \ \mu\mathrm{m}^{2}/\mathrm{s} \enspace, \quad \beta = 36 \text{ s}^{-1} \enspace,\\ \quad \gamma &= 0.2 \text{ s}^{-1} \enspace,\quad p = 0.3 \ \mu\mathrm{m}/\mathrm{s} \, \\ k_{1} &= 0.0718 \text{ s}^{-1} \, \quad \text{ and} \quad k_{2} = \frac{k_{1}}{1.16} = 0.0619 \text{ s}^{-1} \ . \end{array} $$

Gaussian noise with the mean set to zero and a variance whose size is approximately 10% of the maximum intensity is added to the results of the forward simulation. The forward simulation result now replace the goal function that is usually extracted from the experimental FLIP images in the calibration process. The rest of the setup, including the initial guesses on the parameters for the calibration, is identical to the one used for the calibration in Fig. 4. Through the calibration process the misfit function *E* was lowered from 639.4 to 169.7 in 388 iterations with 612 function evaluations which took around 10 h. The calibrated parameters are:

29 $$\begin{array}{*{20}l}  \widetilde{\alpha}&= 16.96 \ \mu\mathrm{m}^{2}/\mathrm{s} \enspace, \quad \widetilde{\beta} = 35.99 \text{ s}^{-1} \enspace,\\ \quad \widetilde{\gamma} &= 0.2002 \text{ s}^{-1} \enspace,\quad \widetilde{p} = 0.3003 \ \mu\mathrm{m}/\mathrm{s} \, \\ \widetilde{k_{1}} &= 0.07182 \text{ s}^{-1} \, \text{ and} \quad \widetilde{k_{2}} = \frac{\widetilde{k_{1}} }{1.16}= 0.06191 \text{ s}^{-1} \ . \end{array} $$

A small error is seen on the fourth digit, which is due to both the Gaussian noise and the size of the stop criterion for the Nelder–Mead algorithm. To determine the sensitivity and robustness of the calibration of the model against parameter variation, the fit to the experimental FLIP data has been repeated for different initial parameter values for both, the semi-permeable and the active membrane transport model (See Additional file 1).

## Discussion

Phase separation and aggregation of polyQ proteins are prominent signs of certain neurodegenerative diseases. Often, protein inclusions of GFP–tagged polyQ proteins are first visible in cells after several days in culture allowing only for studying relatively inert, bright and stable aggregate structures [13, 15]. Thus a key requirement in traditional approaches is that the IBs and similar fluorescent protein aggregates differ in their intensity significantly from the fluorescent protein pool in the surrounding cyto- or nucleoplasm. This, however, limits the analysis to certain inclusion types. Here, we present a new computational approach for inferring diffusion, membrane permeability, and exchange rate constants of GFP–mtHtt between cytoplasm and aggregates of differing brightness directly from experimental FLIP image sequences. Our method allows for detection and dynamic characterization of protein aggregates even in cases, where they are not visible in single image acquisitions.

Using the calibrated reaction–diffusion model, we found that rate constants for exchange of GFP–mtHtt between such large but dim inclusions and the cytoplasm are fast (binding rate constant *k*_1_=0.0718 s^−1^ (Fig. 4) and *k*_1_=0.0111 s^−1^ (Fig. 5) and release rate constant of *k*_2_=0.0619 s^−1^ (Fig. 4) and *k*_2_=0.0109 s^−1^ (Fig. 5). We found similar values previously for the same protein and cell system using a simple multi–compartment model which ignored diffusion and nucleo–cytoplasmic exchange of GFP–mtHtt (i.e. binding rate constant *k*_1_=0.016±0.006 s^−1^ and release rate constant of *k*_2_=0.0127±0.004 s^−1^, mean ± SEM of 6 cells) [7]. From that, we can conclude, that the typical residence time of GFP–mtHtt once bound to cytoplasmic aggregates is on order 16–83 s before being again released and available for free cytoplasmic transport and nucleo–cytoplasmic exchange. Our estimates of intracellular diffusion constants for GFP–mtHtt of $\alpha =\frac {1}{2}(15.9 + 17.6) = 16.75 \ \mu \mathrm {m}^{2}/\mathrm {s}$ are in good agreement with what would be expected for a protein the size of GFP-Q73 (i.e. Stokes radius of *R*≈3.4 nm [9]) in the cytoplasm (i.e. viscosity of $\eta =3.79 \cdot 10^{-9} \frac {\text {kg}}{\mathrm {s} \cdot \mathrm {m}}$ predicts *α*=16.6 according to data from [47]). Supporting that notion is a previous report, which found *α*=18.4±3.3*μ*m^2^/*s* for diffusion of GFP–mtHtt of the same size (i.e., Q73) in the cytoplasm of N2a cells using FRAP [9]. Using an average cytoplasmic diffusion constant of *α*=16.75*μ*m^2^/*s* and the upper estimate of the time constant for binding of 1/(*k*_1_=0.0718 s^−1^)=14 s from our analysis, we conclude that GFP–mtHtt can diffuse on average 30*μ*m away from an aggregate after release before the next binding event takes place. Thus, diffusion is not limiting the aggregation kinetics, which explains, why we found very similar estimates for the binding and dissociation constants as reported here with our previous model which ignored cytoplasmic diffusion altogether [7]. A further point to note is that the IBs in this and our previous study are circular suggesting that they are in a liquid-like state phase-separated from the cytoplasmic pool. This is in line with a recent study [48] and could set a mechanistic basis for the rapid exchange kinetics we observed for GFP-mtHtt between aggregates and cytoplasm. We believe that rapid diffusion and exchange of soluble mtHtt with cytoplasmic inclusions could contribute to the efficient recruitment of other proteins to IBs which further accelerates cellular dysfunction as observed in various studies [6, 14, 49].

We found that a model considering only passive exchange or only active transport of GFP-mtHtt across the nuclear membrane describe the experimental FLIP data almost equally well. The estimated passive permeability is with *p*=0.4 *μ*m/s higher than that of the much smaller GFP [28] suggesting that additionally, active transport mechanisms are at play to facilitate passage of mtHtt across the nuclear membrane. We accounted for active transport of mtHtt by using an additional reactive term at the nucleus-cytoplasm boundary and estimated kinetic rate constants for such a process. Since the equilibrium constant $K_{m}=\frac {k_{cn}}{k_{nc}}$ is only slightly larger than unity, we conclude that transport of GFP-mtHtt is similarly accelerated by cytosolic and nuclear exchange factors, such as importin in the cytoplasm and exportin in the nucleus.

As those components of nucleo-cytoplasmic transport are not explicitly considered in our model, a direct comparison of the rate constants, we obtain to other kinetic models which account for the details of the transport machinery is not possible. However, we could use our active transport model to study the effect of inhibitors or mutations in the NES of mtHtt on its transport in FLIP experiments. In accordance with studies from Truant and colleagues, we find that a slowed nuclear export of huntingtin increases its nuclear accumulation [25, 26]. Using our model, we can additionally predict that slowed nuclear export will fasten the fluorescence loss kinetics of mtHtt in the nucleus and in parallel affect the kinetics of fluorescence loss measured in cytoplasmic IBs in FLIP experiments. Such predictions can be directly tested in future studies.

From the sensitivity test in the Additional file 1, it is clear that it is hard to determine the bleaching constant *β* precisely, as the minimal and maximal *β* found was approximately 16 and 250 s^−1^ for comparable values of the misfit functional (see S.1.1 in Additional file 1). This we see as a consequence of the very powerful laser that bleaches all the fluorescence proteins in the bleaching area. Ignoring all other terms than the bleaching term in (5), the equation simplifies to *c*_*t*_=−*βc*. At the end of the bleaching time *t*=2, with initial value *c*_0_=1, it is seen that the difference between the two solutions for this simple differential equation with *β*=16 and *β*=250 is smaller than 10^−13^. Thus, the change in *β* does not have a significant impact on the solution and may, therefore, be hard to determine. The low sensitivity of the calibration results against changes in *β* may indicate that a better description of the FLIP process necessitates a three-dimensional FLIP model in the future. Indeed, we observed in preliminary experiments that a 3D bleach profile in shape of a double cone is more adequate in modeling 3D FLIP experiments (Hansen et. al. unpublished data).

Our model allows for testing cellular mechanisms underlying observed live-cell FLIP image sequences, but parameter inference from the experimental data is restricted to a few parameters. This is necessary, as otherwise, low parameter sensitivity and model redundancy would follow. For example, only one reaction rate is fitted for all aggregates in the same cell. Each new reaction rate per aggregate would increase the complexity of the calibration process, such that one should have independent evidence for such heterogeneity before extending the model into that direction. For the readers that may want individual reaction mechanics for each aggregate, we suggest to calibrate the parameters *α*,*β*,*γ* and *p* first and then fix these parameters while finding the ones for the aggregates. This can be done under the assumption that the traffic from the aggregates is so small that it would not affect the other parameters.

## Conclusion

Our new computational method allows one to determine diffusion constants, nucleo-cytoplasmic transport parameters and exchange kinetics of polyQ proteins, such as mtHtt, from live-cell FLIP image data. It is the first time, to our knowledge, that all such transport parameters can be inferred in parallel from the full spatiotemporal FLIP intensity profile directly within the cell geometry. Using this new method, we find that polyQ proteins can exchange rapidly between cytoplasm and aggregates and that diffusion of protein monomers is not limiting this exchange process. Furthermore, we show that computational FLIP is an efficient method to detect dim protein aggregates due to their delayed fluorescence loss. Binding and dissociation constants of mtHtt to and from such aggregates are comparable such that the inclusions are hardly visible in single images. Such dim and round aggregates of mtHtt have been recently characterized as being in a liquid-like state, phase separated from the monomeric cytoplasmic pool of the protein [48]. Our computational FLIP approach allows for a systematic study of the properties of such liquid-like aggregates and their transformation towards solid inclusions during the progression of the disease.

Finally, we also model the nucleo-cytoplasmic transport of GFP-mtHtt and show that mutated Htt shuttles rapidly across the nuclear membrane, likely by Ran-mediated active transport. Nuclear accumulation precedes the formation of aggregates and IBs of mtHtt in the nucleus, which likely impairs transcription of essential genes in the affected cells [14]. Our new method can be employed in the future to systematically study the effect of mtHtt aggregation on its transport across the nuclear membrane. Thus our method sets the stage for a systematic exploration of how the aggregation process affects the nucleo-cytoplasmic permeability of polyQ proteins. Our new approach is widely applicable to quantify protein dynamics in cellular inclusions of various disease models.

## Additional file

Additional file 1 A discontinuous Galerkin model for fluorescence loss in photobleaching of intracellular polyglutamine protein aggregates. (PDF 215 kb)
