# Supplementary material for: A discontinuous Galerkin model for fluorescence loss in photobleaching of intracellular polyglutamine protein aggregates
Source: BMC Biophys. 2018 Nov 29;11:7. doi: 10.1186/s13628-018-0046-0 (PMC6264036; doi:10.1186/s13628-018-0046-0)
Supplement: Supplementary file 1 — A discontinuous Galerkin model for fluorescence loss in photobleaching of intracellular polyglutamine protein aggregates. (PDF 215 kb) [file 13628_2018_46_MOESM1_ESM.pdf]

A discontinuous Galerkin model for fluorescence loss in photobleaching of intracellular polyglutamine protein aggregates

Christian V. Hansen[1], **Hans J. Schroll**[2], **Daniel Wüstner**[3]

[1,2,3] *University of Southern Denmark, Campusvej 55, DK-5230 Odense M, Denmark*
[1] *Department of Mathematics and Computer Science* `cvh@imada.sdu.dk`
[2] *Department of Mathematics and Computer Science* `achim@imada.sdu.dk`
[3] *Department of Biochemistry and Molecular Biology* `wuestner@bmb.sdu.dk`

S Supplementary Information

S .1 Sensitivity analysis

A brief sensitivity analysis for both the active and permeable membrane model is here presented. For both models 30 calibrations with different initial guesses where made. For the active membrane model 25 of the calibrations succeed, whereas 24 for the permeable membrane succeed i.e. the Nelder-Mead simplex algorithm converged towards a minima. The full dataset can be found in Section S .1.1, where Table S1 and S3 shows the normal random chosen initial values and Table S2 and S4 shows the calibration results. The initial guesses are random numbers from the normal distribution $N(\mu, \sigma)$ with means μ as $\mu_\alpha = 25$, $\mu_\beta = 20$, $\mu_\gamma = 0.5$, $\mu_{(k_1)} = 0.001$ and either $\mu_p = 0.05$ or $\mu_{(k_{nc})} = 0.05$ and the standard deviation $\sigma = \frac{\mu}{2}$ for each corresponding parameter.

The calibrated parameters from Table S2 and S4 are plotted in Figure S1 and S2. k_2 is not shown as k_1 and k_2 are proportional. Neither is k_{nc} as k_{nc} and k_{cn} are proportional.
In Figure S1 the results from Table S2 with the active membrane model are manually put into three groups for better visualization. 16 points are red, 6 are blue and 3 are green.
In Figure S2 the results from Table S4 are manually put into two groups for better visualization. 16 points are red and 8 are blue.

Looking at the variances for both models, it is clear that it is hard to determine the bleaching constant β precisely. This is a consequence of the very powerful laser that bleaches all the fluorescence proteins in the bleaching area. Ignoring all terms in the PDE except for the bleaching term in the bleaching area the equation simplifies to $c_t = -\beta c$. Calculating the exact solution for the minimal and maximal β found i.e. 16 and 250, at the end of the bleaching time $t = 2$, with initial value $c_0 = 1$, it is seen that the difference between the two solutions are smaller than 10^{-13}.

Looking at the results from Table S2 and S4, and especially the mean of the misfit functional E, it is possible to see that there is no significant difference on how well the two models behave.

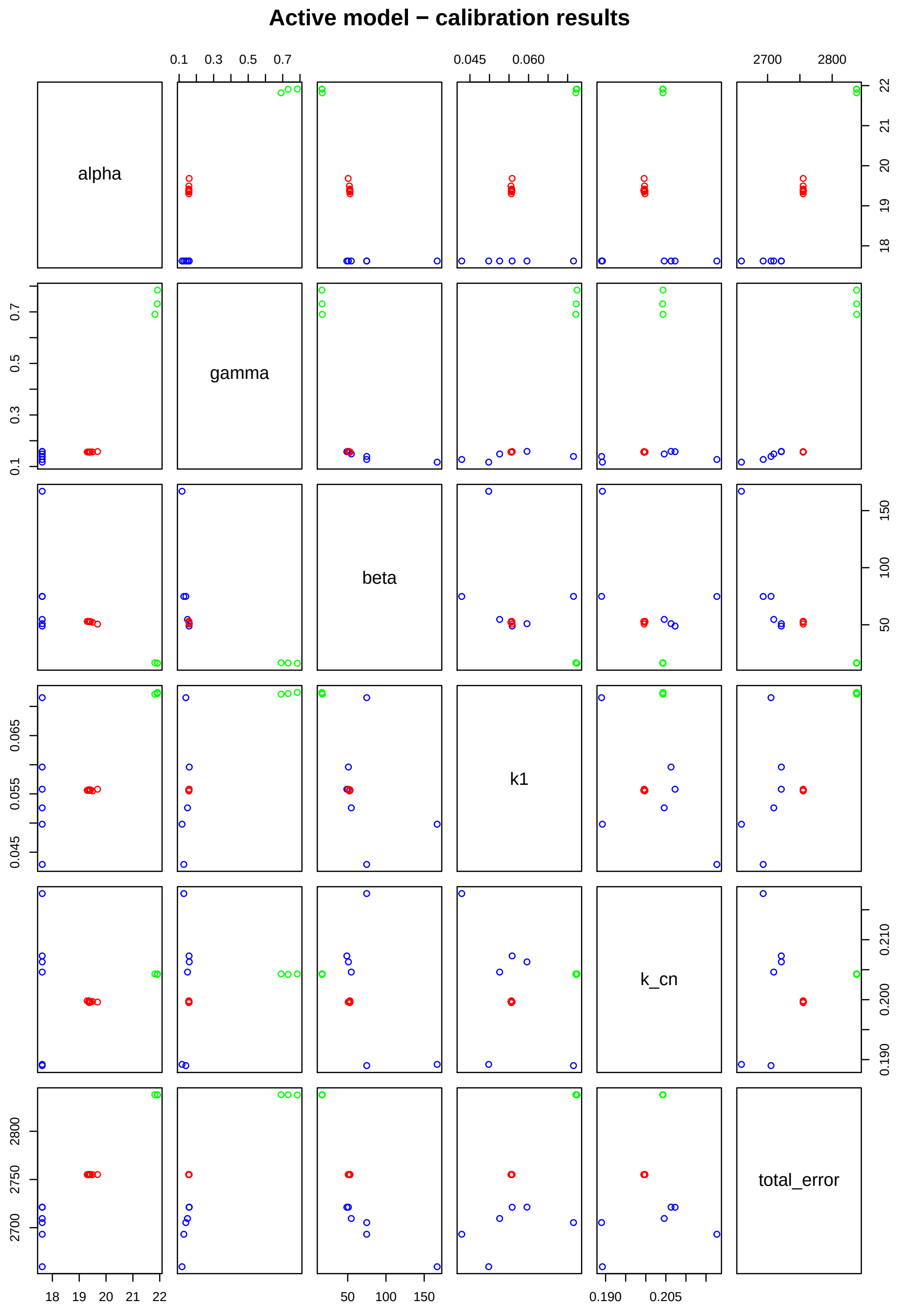

Figure S1: Result of 25 calibrations with the active membrane model. They where manually grouped into three groups. 16 points are red, 6 are blue and 3 are green for better visualization.

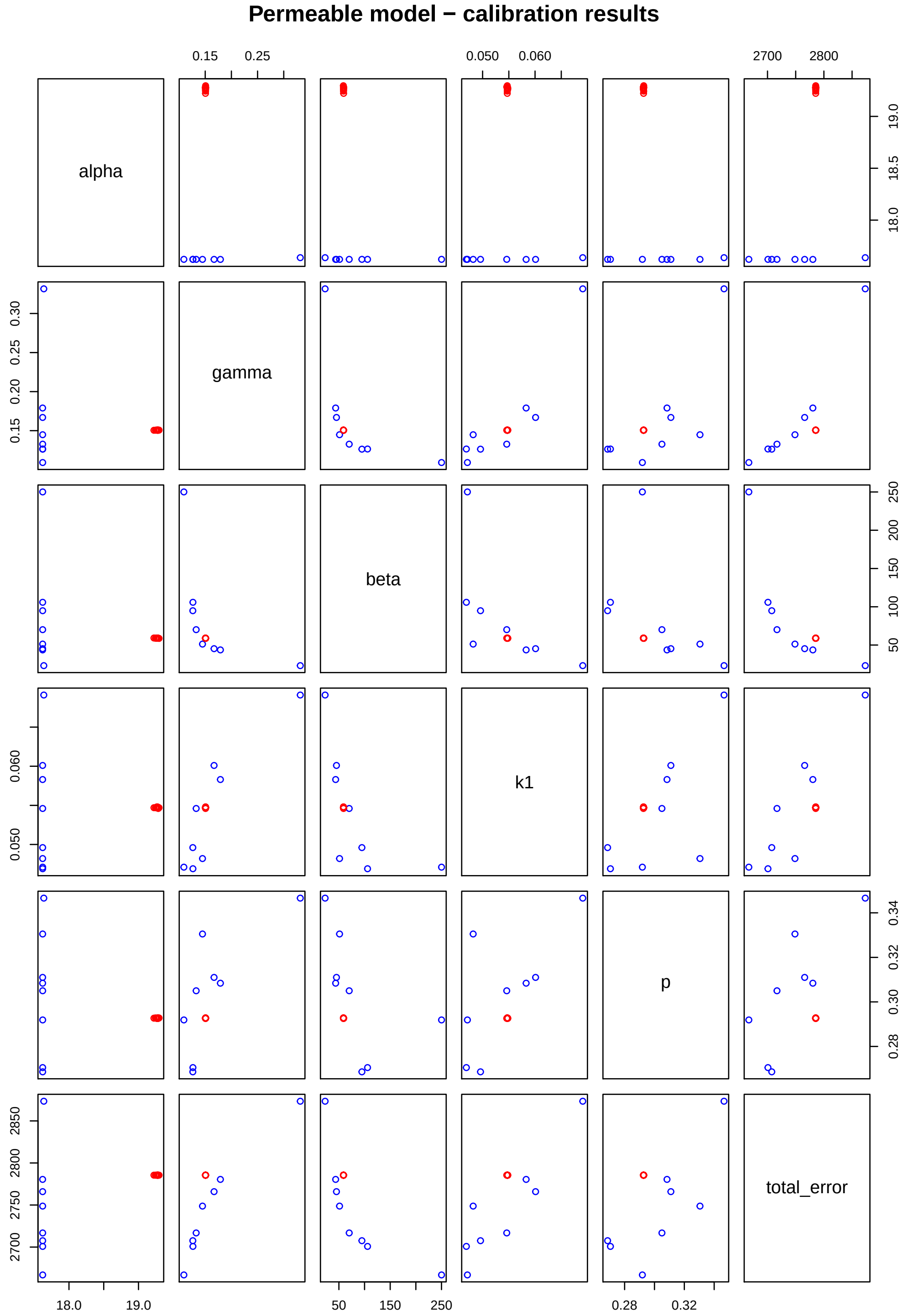

Figure S2: Result of 24 calibrations with the permeable membrane model. They where manually grouped into two groups. 16 points are red and 8 are blue for better visualization.

S .1.1 Data

Table S1: Initial values for 25 different calibrations with the model that uses active transport across the membrane.

Run index	α_0	γ_0	β_0	$(k_2)_0$	$(k_1)_0$	$(k_{nc})_0$	$(k_{cn})_0$
1	30.1454	0.6313	27.2455	0.0010	0.0012	0.0369	0.0465
2	31.6865	0.4345	14.0790	0.0016	0.0018	0.0375	0.0473
3	3.9519	0.2643	9.8192	0.0016	0.0019	0.0821	0.1035
4	34.5325	0.8521	26.2761	0.0015	0.0018	0.0665	0.0838
5	22.5407	0.4860	16.9180	0.0018	0.0021	0.0457	0.0577
6	30.1162	0.3541	9.7417	0.0012	0.0014	0.0901	0.1136
7	35.4624	0.3509	31.8381	0.0015	0.0017	0.0418	0.0527
8	7.9731	0.7660	23.9820	0.0003	0.0004	0.0608	0.0766
9	28.6723	0.6164	23.6914	0.0007	0.0008	0.1131	0.1426
10	18.8302	0.2716	24.3397	0.0008	0.0009	0.0663	0.0835
11	28.3472	0.1971	17.0505	0.0010	0.0012	0.0482	0.0607
12	27.2461	0.2616	23.9108	0.0017	0.0020	0.0986	0.1243
13	23.6419	0.3182	8.0295	0.0014	0.0017	0.0300	0.0378
14	12.9890	0.5421	33.8854	0.0006	0.0007	0.0993	0.1251
15	13.3557	0.5753	21.9863	0.0004	0.0005	0.0384	0.0484
16	35.8313	0.5589	14.2522	0.0021	0.0025	0.0187	0.0235
17	19.7588	0.5854	16.1501	0.0008	0.0009	0.1253	0.1579
18	39.6636	0.5365	2.6587	0.0006	0.0007	0.0705	0.0889
19	27.3121	0.3433	24.8877	0.0003	0.0004	0.0459	0.0578
20	29.1804	0.4639	23.8538	0.0003	0.0004	0.0362	0.0456
21	5.1277	0.9135	8.5255	0.0011	0.0013	0.0659	0.0830
22	32.7218	0.2979	40.9214	0.0008	0.0009	0.1385	0.1746
23	48.0505	0.6186	31.8555	0.0009	0.0010	0.0789	0.0994
24	41.4855	0.4281	30.4122	0.0014	0.0016	0.0700	0.0882
25	4.8098	0.5489	26.7342	0.0011	0.0013	0.0909	0.1146

Table S2: Calibration results of 25 different calibrations with different initial values for the model that uses active transport across the membrane.

Run index	α	γ	β	k_2	k_1	k_{nc}	k_{cn}	E
1	19.3906	0.1563	52.7609	0.0479	0.0556	0.1996	0.2515	2755.0601
2	19.3621	0.1563	52.8479	0.0480	0.0557	0.1996	0.2516	2755.0606
3	19.3648	0.1565	52.6055	0.0480	0.0556	0.1998	0.2517	2755.0617
4	21.8198	0.6906	16.7431	0.0621	0.0721	0.2043	0.2574	2837.9487
5	19.3769	0.1563	52.8554	0.0479	0.0556	0.1996	0.2516	2755.0601
6	19.3491	0.1563	52.8391	0.0480	0.0557	0.1996	0.2515	2755.0606
7	19.3794	0.1563	52.7536	0.0480	0.0556	0.1996	0.2516	2755.0601
8	19.3735	0.1562	52.8784	0.0480	0.0556	0.1996	0.2516	2755.0604
9	19.2987	0.1563	52.9965	0.0479	0.0556	0.1998	0.2518	2755.0614
10	17.6214	0.1587	50.9524	0.0514	0.0596	0.2063	0.2600	2721.3123
11	17.6215	0.1486	54.6873	0.0454	0.0526	0.2046	0.2578	2709.5312
12	19.3797	0.1562	52.8562	0.0479	0.0556	0.1995	0.2515	2755.0605
13	19.3648	0.1563	52.8604	0.0480	0.0556	0.1997	0.2516	2755.0603
14	19.3902	0.1563	52.7993	0.0480	0.0556	0.1996	0.2516	2755.0603
15	17.6217	0.1273	74.7882	0.0370	0.0429	0.2177	0.2744	2693.2044
16	19.4944	0.1566	52.1836	0.0479	0.0555	0.1997	0.2517	2755.0666
17	17.6218	0.1390	74.8745	0.0616	0.0715	0.1890	0.2382	2705.2985
18	21.9078	0.7311	16.5141	0.0623	0.0722	0.2042	0.2574	2837.9250
19	19.4148	0.1563	52.7042	0.0480	0.0556	0.1997	0.2517	2755.0606
20	17.6214	0.1575	48.8553	0.0481	0.0558	0.2073	0.2612	2721.2092
21	21.9158	0.7845	16.2592	0.0624	0.0724	0.2043	0.2575	2837.7083
22	19.6822	0.1581	50.6054	0.0481	0.0558	0.1996	0.2516	2755.1334
23	19.3530	0.1563	52.8682	0.0480	0.0556	0.1996	0.2516	2755.0602
24	17.6218	0.1169	166.9476	0.0430	0.0498	0.1892	0.2385	2659.5034
25	19.4138	0.1564	52.6835	0.0480	0.0557	0.1996	0.2515	2755.0604
Mean:	19.2704	0.2223	54.5088	0.0496	0.0576	0.2008	0.2531	2752.1875
Variance:	1.5475	0.0377	746.1381	0.0000	0.0001	0.0000	0.0000	1673.0275

Table S3: Initial values for 24 different calibrations with the permeable membrane model.

Run index	α_0	γ_0	β_0	$(k_2)_0$	$(k_1)_0$	p_0
1	19.4641	0.4234	27.3758	0.0015	0.0018	0.0610
2	32.8348	0.3342	17.3334	0.0012	0.0013	0.0384
3	33.3718	0.7470	13.6322	0.0010	0.0011	0.0247
4	16.8856	0.4942	25.8209	0.0008	0.0010	0.0425
5	20.0768	0.3619	31.1824	0.0013	0.0015	0.0630
6	5.5973	0.0960	16.2186	0.0015	0.0018	0.0593
7	40.1895	0.3723	29.6357	0.0015	0.0017	0.0519
8	31.2136	0.6484	17.5033	0.0007	0.0009	0.0440
9	33.9463	0.4685	22.0034	0.0003	0.0003	0.0674
10	34.3129	0.5192	19.2424	0.0009	0.0011	0.0223
11	42.3781	0.1957	23.4124	0.0012	0.0014	0.0707
12	24.9924	0.5396	17.8099	0.0009	0.0010	0.0674
13	28.5822	0.8035	24.7509	0.0008	0.0009	0.0194
14	16.5359	0.3993	7.3709	0.0009	0.0010	0.0273
15	15.2361	0.5695	0.0433	23.3684	0.0007	0.0008
16	23.6460	0.6601	0.0431	15.6216	0.0008	0.0009
17	23.8558	0.4129	0.0322	19.8913	0.0014	0.0016
18	24.3310	0.5775	0.0686	20.7471	0.0015	0.0017
19	25.7374	0.5217	0.0548	23.7272	0.0017	0.0020
20	26.9565	0.8108	0.0416	14.2164	0.0010	0.0012
21	27.9582	0.4257	0.0626	20.1761	0.0011	0.0013
22	29.5311	0.5823	0.0389	27.3953	0.0011	0.0013
23	30.3369	0.2317	0.0322	13.9155	0.0009	0.0010
24	31.5754	0.5490	0.0487	26.3221	0.0011	0.0012

Table S4: Calibration results of 24 calibrations with different initial values for the permeable membrane model.

Run index	α	γ	β	k_2	k_1	p	E
1	19.2775	0.1507	58.8323	0.0472	0.0547	0.2927	2785.4905
2	17.6219	0.1092	250.0607	0.0406	0.0471	0.2919	2666.8877
3	17.6217	0.1265	105.8486	0.0404	0.0469	0.2705	2700.7931
4	19.2624	0.1506	58.9730	0.0472	0.0547	0.2926	2785.4903
5	19.2632	0.1507	58.8299	0.0472	0.0548	0.2927	2785.4908
6	19.2841	0.1506	58.9856	0.0471	0.0546	0.2926	2785.4905
7	17.6211	0.1790	43.6408	0.0502	0.0583	0.3084	2780.4535
8	19.2772	0.1506	58.9316	0.0472	0.0547	0.2928	2785.4903
9	19.2788	0.1507	58.8871	0.0471	0.0547	0.2927	2785.4905
10	17.6213	0.1669	45.2487	0.0518	0.0601	0.3110	2765.9007
11	19.2222	0.1505	59.1915	0.0472	0.0547	0.2927	2785.4910
12	19.2795	0.1505	59.0516	0.0472	0.0547	0.2926	2785.4911
13	19.2829	0.1506	58.8948	0.0472	0.0547	0.2928	2785.4899
14	17.6215	0.1448	51.2572	0.0416	0.0482	0.3305	2748.7236
15	19.2681	0.1506	58.9524	0.0472	0.0547	0.2926	2785.4905
16	19.2956	0.1507	58.6897	0.0471	0.0547	0.2928	2785.4913
17	17.6217	0.1327	70.1003	0.0470	0.0546	0.3050	2716.8113
18	19.2742	0.1506	58.9223	0.0472	0.0548	0.2926	2785.4902
19	17.6381	0.3316	23.1079	0.0595	0.0691	0.3466	2873.3972
20	19.2753	0.1507	58.9315	0.0471	0.0547	0.2928	2785.4904
21	17.6218	0.1263	94.8552	0.0427	0.0496	0.2686	2707.5802
22	19.2518	0.1506	59.0140	0.0471	0.0547	0.2927	2785.4900
23	19.2434	0.1506	58.9936	0.0472	0.0547	0.2928	2785.4907
24	19.2783	0.1506	58.8846	0.0472	0.0547	0.2927	2785.4901
Mean:	18.7210	0.1553	67.7952	0.0470	0.0546	0.2965	2772.0165
Variance:	0.6285	0.0016	1740.2040	0.0000	0.0000	0.0003	1615.5860
